# Selective enrichment of founding reproductive microbiomes allows extensive vertical transmission in a fungus-farming termite

**DOI:** 10.1098/rspb.2023.1559

**Published:** 2023-10-18

**Authors:** Veronica M. Sinotte, Justinn Renelies-Hamilton, Sergio Andreu-Sánchez, Mireille Vasseur-Cognet, Michael Poulsen

**Affiliations:** ^1^ Section for Ecology and Evolution, Department of Biology, University of Copenhagen, 2100 Copenhagen East, Denmark; ^2^ Center for Evolutionary Hologenomics, GLOBE Institute, University of Copenhagen, 1350 Copenhagen K, Denmark; ^3^ Department of Paediatrics, University Medical Centre Groningen, University of Groningen, 9700 RB Groningen, The Netherlands; ^4^ UMR IRD 242, UPEC, CNRS 7618, UPMC 113, INRAe 1392, Paris 7 113, Institute of Ecology and Environmental Sciences of Paris, Bondy, France; ^5^ Institut National de la Santé et de la Recherche Médicale, Paris, France

**Keywords:** symbiosis, Macrotermitinae, social insects, superorganism, coevolution, microbiota

## Abstract

Mutualistic coevolution can be mediated by vertical transmission of symbionts between host generations. Termites host complex gut bacterial communities with evolutionary histories indicative of mixed-mode transmission. Here, we document that vertical transmission of gut bacterial strains is congruent across parent to offspring colonies in four pedigrees of the fungus-farming termite *Macrotermes natalensis*. We show that 44% of the offspring colony microbiome, including more than 80 bacterial genera and pedigree-specific strains, are consistently inherited. We go on to demonstrate that this is achieved because colony-founding reproductives are selectively enriched with a set of non-random, environmentally sensitive and termite-specific gut microbes from their colonies of origin. These symbionts transfer to offspring colony workers with high fidelity, after which priority effects appear to influence the composition of the establishing microbiome. Termite reproductives thus secure transmission of complex communities of specific, co-evolved microbes that are critical to their offspring colonies. Extensive yet imperfect inheritance implies that the maturing colony benefits from acquiring environmental microbes to complement combinations of termite, fungus and vertically transmitted microbes; a mode of transmission that is emerging as a prevailing strategy for hosts to assemble complex adaptive microbiomes.

## 1. Introduction

Associations with microbiomes unequivocally impact the ecology and evolution of animal hosts. From humans to insects, animals consistently engage with microbial partners to reap synergistic metabolic, immune and defensive benefits [1–3]. Vertical transmission, the process by which microbes are passed from parents to offspring, ensures inheritance of beneficial microbes [4] and provides an initial inoculum that directs microbiome assembly [5]. Over evolutionary time, consistent vertical transmission aligns the reproductive interests of hosts and microbes and is predicted to favour mutualism and host–symbiont co-adaptations [6,7]. In the most intimate of mutualisms, hosts integrate bacteria within cells and transmit them via their gametes (eggs) [8]. More commonly, animals transmit complex microbiomes externally at birth or early in development, securing microbial inheritance while allowing for the acquisition of environmental microbes via horizontal transmission [9,10].

Evolutionary histories between social insects and bacterial symbionts [11–13] imply vertical transmission and host adaptations that secure some extent of inheritance. In superorganismal social insects, such as ants, corbiculate bees and Termitidae termites, these adaptations are predicted to occur at the colony level, which natural selection acts on as an individual [14]. Superorganismal colony life begins with winged (alate) reproductives that leave their parental colony to found offspring colonies. Vertical transmission via founding reproductives should thus allow inheritance of symbiotic microbes between generations. Offspring colony workers then sustain a stable symbiotic community through trophallaxis [15–17], and microbes can be passed to new reproductives before the colony life cycle begins again. Indeed, founding reproductives carry microbiota from their parental colonies in ants [18,19], bees [20] and termites [21,22]. However, the complete process of vertical transmission of gut microbes from parent colonies via reproductives to offspring colonies has not been documented for any superorganism. A holistic assessment should elucidate transmission on proximate timescales and traits that optimize microbial inheritance.

Termites are valuable models to examine the specificity and mechanisms of vertical transmission because of their 150 Myr long association with beneficial and complex gut microbiomes [23] that have co-adapted to serve digestive roles [24,25]. Co-cladogenesis of termite hosts with bacterial and protist symbionts indicates predominant vertical transmission punctuated by horizontal transmission from other hosts or the environment [11,26–28]. Yet, the transmission between colony generations, which in part drives these evolutionary patterns, is not well understood. Founding reproductives flying from their parental colonies have many of the core microbial genera found in workers [21,22,29]. Separate works have found that male and female founding reproductives (king and queen) transfer a large portion of their microbiome to workers [30,31]. Assessing the complete process of transmission is necessary to clarify (i) the extent of vertical transmission, (ii) what microbial taxa persist across generations and (iii) how founding reproductives or workers impact the transmitted microbial lineages.

Here, we assess vertical transmission from parent to offspring colonies for the first time by tracking bacterial strains across pedigrees of the fungus-farming termite *Macrotermes natalensis*. As in other termites, fungus-farming termites (Termitidae: Macrotermitinae) host a specific [32], consistent [33,34], and co-evolved gut microbiome [35,36]. Given the consistent complexity of the microbiome and evolutionary patterns of mixed-mode transmission [11,27], we first hypothesized that a subset of the colony microbiome would be vertically transmitted and contain a conserved diverse set of microbes. Secondarily, we hypothesized that transmission to founding reproductives would have a greater impact on the inherited microbiome than subsequent transmission to offspring workers. As reproductives represent the sole opportunity to transmit the microbiome that then persists in millions of workers and soldiers, they should be under strong selection to optimize transmission. To test these hypotheses, we quantified vertical transmission first from parental colonies to reproductives and then from reproductives to offspring colonies. We subsequently predicted host factors and microbial interactions that facilitate vertical transmission and confirmed their impacts with a random forest (RF) model.

## 2. Results

### (a) The termite superorganism: a model system for vertical transmission

**Figure 1.**
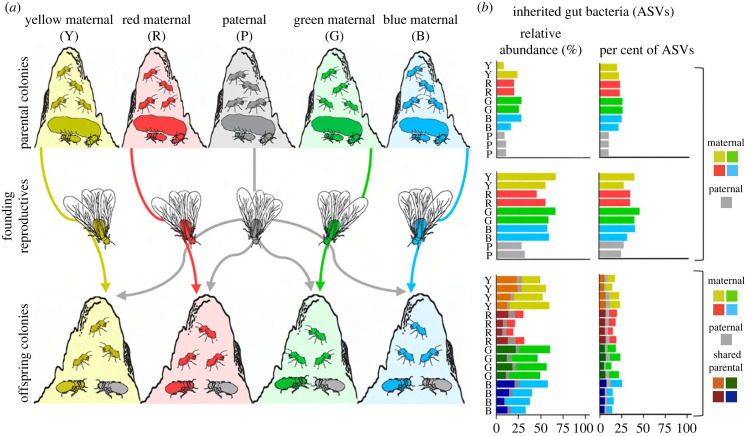
Biparental inheritance of bacteria across independent termite pedigrees. (*a*) We used a pedigree design crossing four maternal colonies with a paternal colony to assess vertical transmission of ASVs from parental colonies, through founding reproductives, to offspring colonies. (*b*) A substantial portion of the vertically transmitted microbiome was detected in parental colonies (top), founding reproductives (centre) and offspring colonies (bottom) (electronic supplementary material, table S1). Inheritance was biparental, resulting in ASVs that were uniquely maternal, paternal or shared by parental lineages. The extent of paternal inheritance (grey) is likely an underestimate because of our sampling (see Materials and methods). Bars represent community analyses from five to 10 pooled guts (electronic supplementary material, table S2). Only workers in parental and offspring colonies are plotted as they play the primary role in maintaining bacterial diversity and loads (electronic supplementary material, figure S1).

To first assess the extent of vertical transmission, we tracked bacterial strains across colony generations. We used amplicon sequencing of the V3–V4 region of the 16S *rRNA* bacterial gene to track individual amplicon sequence variants (ASVs) from parental colonies through founding reproductives to offspring colonies in four independent pedigrees (figure 1*a*). To establish pedigrees, founding queens from four independent maternal colonies were crossed with founding kings from a paternal colony to establish offspring colonies [37]. This allowed us to identify microbes transmitted within pedigrees, indicative of vertical transmission [38,39], and biparental contributions to inheritance. Offspring colonies were sampled when three months old, prior to acquisition of the fungal cultivar.

We invoked a strict definition of inheritance to assess the consistency of vertical transmission. ASVs were defined with stringent parameters (see Materials and methods), and vertical transmission required an ASV to be present in one of the two parental colonies, in all founding reproductive samples from that parental colony, and in resulting offspring colonies. Although inherited microbes may be hosted by any termite in the colony, we focused our analyses on workers and founding reproductives that, respectively, house and transmit most of the colony microbiome [31,40] (electronic supplementary material, figure S1). Five to 10 guts were pooled in each sample, which may reduce the variation present between individual termites yet be more representative of the colony microbiome. Regardless of microbial load and composition, the sequencing effort sufficiently captured ASV diversity across castes (electronic supplementary material, figure S2).

### (b) Extensive and consistent vertical transmission of a diverse microbiome

The microbiome transmitted from parent to offspring colonies was, as predicted, extensive and congruent across pedigrees (figure 1*b*). The termites key to microbial inheritance—parental colony workers, founding reproductives, and offspring colony workers—maintained high relative abundances of vertically transmitted ASVs (figure 1*b*). Parental colony workers transmitted 21.2% relative abundance and 24.1% of ASVs in their microbiome to the founding reproductives and ultimately offspring colonies. Founding reproductives hosted the greatest abundance and per cent of vertically transmitted microbes, averaging 57.6% of the relative abundance and 38.6% ASVs. Offspring colony workers hosted vertically transmitted ASVs that made up 43.9% of microbial abundance and 18.7% of all ASVs. The specific number of vertically transmitted ASVs may have been impacted by the stringent definition of ASVs and the pooling of guts (electronic supplementary material, tables S1 and S2). Our findings extend recent insights from another fungus-farming termite, *Macrotermes subhyalinus,* where vertical transmission from founding reproductives to offspring colonies was documented [31]. Our inclusion of parental colonies, more stringent taxonomic classification, and genetic replicates through the pedigree design potentially led us to quantify less vertical transmission, previously found to be 73% of the relative abundance and 60% OTU diversity [31]. Biparental contributions to vertical transmission were evident from offspring colony microbiomes containing ASVs that were uniquely maternal, uniquely paternal or shared by both parental colonies (figure 1), consistent with findings on protist inheritance in non-Termitidae termites [22,41]. Paternal contributions were likely to be greater than those detected because delayed sampling of paternal colony workers could have shifted microbiome composition (see Materials and methods). We thus focused subsequent analyses on maternal vertical transmission. Termite outbreeding may thus promote complementarity between maternal and paternal microbiomes, resulting in both redundancy and variation in the offspring microbiome.

**Figure 2.**
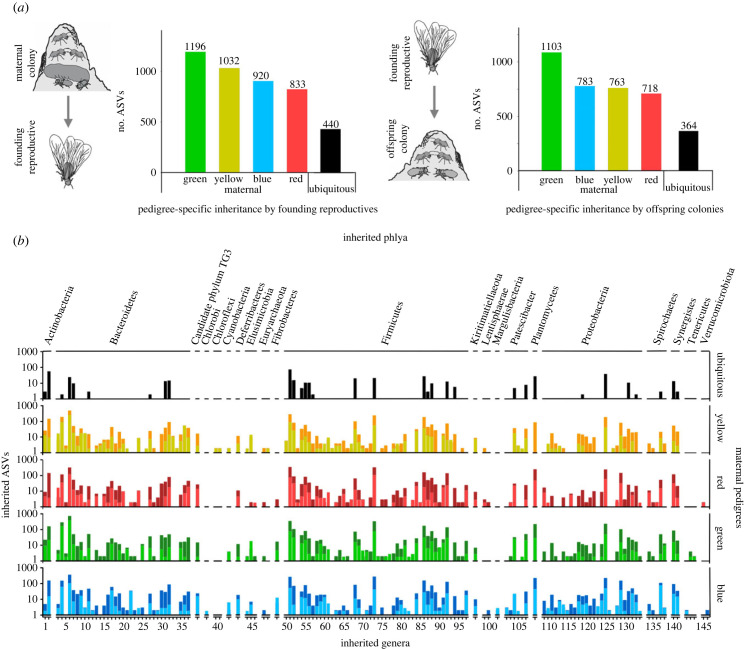
Consistent vertical transmission of bacterial diversity across pedigrees. (*a*) Many ASVs were exclusively inherited in one of the four maternal pedigrees (coloured), indicative of vertical transmission. ASVs transmitted from maternal colonies to founding reproductives (i) were largely retained in subsequent transmission to offspring colony workers (ii). Pedigree-specific inheritance exceeded the number of ASVs that were ubiquitous (black) or shared among pedigrees (electronic supplementary material, figure S3). (*b*) Similarity in the taxonomic signal across pedigrees implies consistency in inheritance at the population level, with on average 95.2% of genera shared by any two pedigrees (electronic supplementary material, figure S3). Pedigree-specific ASVs are indicated in light-coloured bars and generally inherited ASVs in dark bars (electronic supplementary material, table S3).

We then assessed the specificity of the microbiome inherited by quantifying the number of ASVs that were uniquely inherited or shared by individual maternal pedigrees. Each maternal pedigree on average transmitted almost 1000 unique ASVs to founding reproductives, of which 84% were maintained in offspring colonies (figure 2*a*). By contrast, only 364 ASVs were ubiquitous to all pedigrees (figure 2*a*), while others were shared by two or three pedigrees (electronic supplementary material, figure S3A and B). Ultimately, the observed pedigree specificity is an estimate because ASVs themselves do not reflect true bacterial strain diversity [42]. However, this is unproblematic for comparisons of the extent and consistency of transmission [9,39], and is a robust indicator of extensive vertical transmission [38,39].

Evaluating the taxonomic consistency across pedigrees revealed that colonies inherit a diverse and predictable set of microbes. Genus-level compositions of the inherited microbiomes were nearly identical across the independent pedigrees (figure 2*b*). Consequently, 85 genera were inherited by all pedigrees, with any two pedigrees sharing on average 95.2% inherited bacterial genera (electronic supplementary material, figure S3). Pedigree-specific ASVs accounted for many of these genera (electronic supplementary material, table S3), and ASV diversity within genera was also comparable (figure 2*b*). Thus, pedigrees inherit a diverse and congruent set of microbes, including multiple taxa that are consistently associated with fungus-farming termites [31,34], implying that offspring colonies have predictable access to co-adapted symbionts.

### (c) Selective enrichment of founding reproductive microbiomes secures transmission

Founding reproductives are the only avenue for offspring colonies to obtain gut microbes that are absent from the environment during early life. The richness, abundance and consistency of vertically transmitted gut microbes imply that reproductives transmit a specific and optimal set of bacteria (figures 1*b* and 2*a*). In other termites, the relative abundance of protist OTUs in parental workers increases the chance of transmission to founding reproductives [22]. Therefore, the bacteria endowed in founding reproductives may be based on abundance in workers, or reproductives may be adapted to selectively transmit a set of key microbial symbionts.

**Figure 3.**
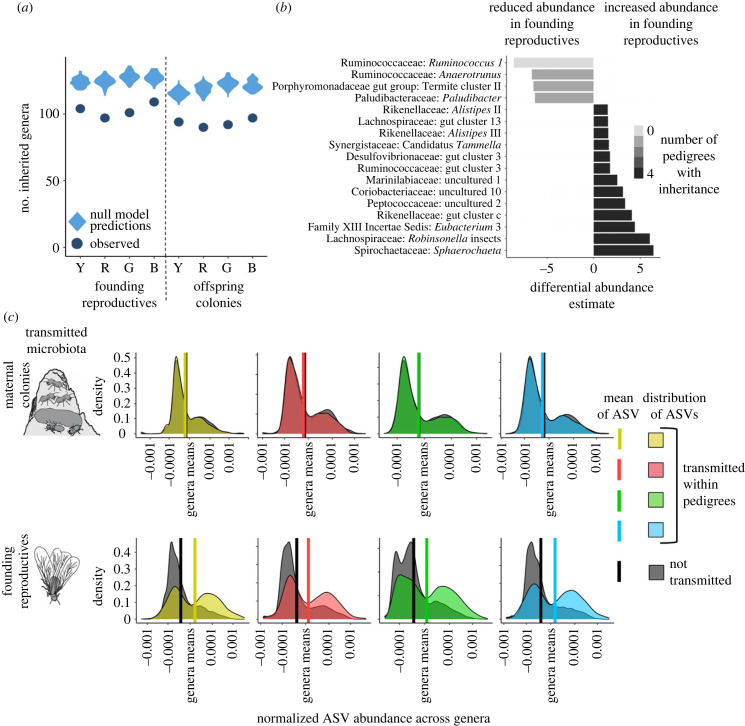
Founding reproductives are endowed with a specific inherited gut microbiome. (*a*) Maternal founding reproductives and offspring colony workers inherited a specific set of bacterial genera that was lower than predicted by the null model (permutation tests: *p* < 0.0001, for each pedigree). (*b*) Vertical transmission endows founding reproductives with an increased abundance of inherited microbial genera. Genera that significantly decreased in abundance were infrequently transmitted, while genera that increased in abundance were always inherited (electronic supplementary material, table S3 and table S4). (*c*) The abundance of ASVs within genera in maternal colonies had a slight negative impact on the chance of vertical transmission (top; general linear mixed model (LMM): *F*_1,48018_ = 10.26, *t* = − 3.20, *p* = 0.0014). By contrast, ASV abundance in founding reproductives significantly improved transmission to offspring colonies (bottom; LMM: *F*_1,40329_ = 2665, *t* = 51.62, *p* < 0.0001). ASV abundances are normalized by the mean abundance within genera (0 on the *x*-axis), and densities are log-transformed for visualization (see Materials and methods). Vertical bars indicate means for transmitted (coloured) and non-transmitted (black) ASVs (electronic supplementary material, table S5).

By comparing a null model to observed inheritance, we elucidated that founding reproductives selectively transmit non-random microbial assemblies. The null model considers an ASV's probability of vertical transmission based on its abundance within maternal colony workers. Thus, it selects the same number of ASVs that were observed to be transmitted from the maternal colony to founding reproductives or founding reproductives to the offspring colony weighted by their abundance in the maternal colony (see Materials and methods). Comparison of the null model to observed transmission revealed that significantly fewer genera were inherited by founding reproductives than predicted by the model (figure 3*a*; permutation tests: *p* < 0.0001, for each pedigree). Most genera in reproductives were inherited by offspring colonies, such that offspring worker communities also contained fewer genera than predicted by the null model (permutation tests: *p* < 0.0001, for each pedigree). Inheritance is thus a product of selection of a non-random subset of microbial genera, implying adaptations that ensure inheritance of specific microbes.

We went on to test whether transmission to founding reproductives impacts the relative abundance of inherited bacterial genera. Differential abundance analysis between maternal colony workers and founding reproductives revealed four genera that were always less abundant in reproductives, which were either never inherited or only inherited in one pedigree (figure 3*b*). By contrast, 13 genera that increased in abundance in founding reproductives completed vertical transmission across all pedigrees (figure 3*b*; electronic supplementary material, table S4)*.* These genera thus appear critical for hosts, consistent with findings that several have diversified with termite hosts [11,27] and are members of the core microbiome [34]. The subsequent transfer to offspring colonies did not drastically alter the inherited microbiome, but some inherited genera either significantly increased or decreased in relative abundance (electronic supplementary material, figure S4 and table S4).

Founding reproductives also shape the ASV-level composition of the inherited microbiome. If vertical transmission was random and abundance driven, ASVs with higher abundance within genera should have a greater chance of transmission. This is not the case for transmission from maternal colony workers to founding reproductives, as increased ASV abundance in maternal colonies significantly reduced the probability of transmission to reproductives (figure 3*c*). Thus, reproductives appear to secure a diverse inoculum of ASVs from key genera rather than allowing competitive exclusion to drive transmission. However, ASV abundance in founding reproductives positively impacted whether ASVs would establish within offspring colony workers (figure 3*c*; electronic supplementary material, table S5). Vertically transmitted microbes that become abundant in founding reproductives thus successfully establish in offspring colonies.

### (d) Vertical transmission grants inherited microbes priority

Founding reproductives vector inherited microbiomes to secure emerging functions of the workers, who perform digestion and provide defence for the colony. Priority effects, in which early-arriving microbes dictate community assembly [43], could both enhance establishment of inherited microbes and later shape the uptake of beneficial non-inherited microbes in the colony microbiome.

**Figure 4.**
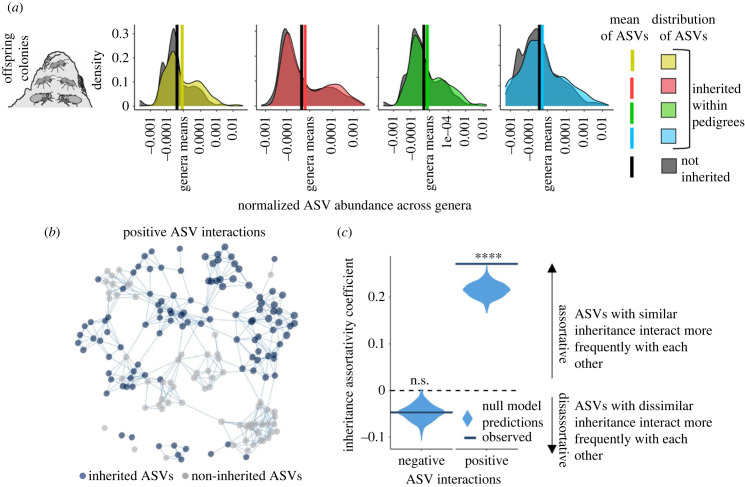
Vertical transmission may grant bacteria priority over horizontally transmitted microbiota. (*a*) The higher abundances of vertically transmitted ASVs observed in founding reproductives (figure 3*c*) persist in offspring colony workers, maturing reproductives and larvae (electronic supplementary material, figure S5). In these workers, ASV abundance significantly relates to whether a strain was inherited or not (*F*_1,20250_ = 116.8, *t* = 10.81, *p* < 0.0001). (*b*) The offspring worker microbiome network revealed that inherited ASVs frequently co-occurred, as did non-inherited ASVs, while co-occurrences of inherited and non-inherited ASVs were less frequent. This assortative clustering likely reflects priority effects because co-occurring inherited ASVs may direct the establishment of non-inherited ASVs. To clarify assortative patterns, the visualized network includes ASVs with positive interactions with at least six others. (*c*) Assortativity of the positive network, based on inheritance, was significantly greater than predicted by our abundance-driven null model (permutation test: *p* < 0.0001; assortativity coefficients: observed network = 0.2714, null model = 0.2150 ± 0.0153). The network of ASVs with negative interactions, where the presence of an ASV was associated with the absence of another, was marginally disassortative but not significantly different from the random null model (permutation test: *p* = 0.6300; assortativity coefficients: observed network = − 0.0467, null model = − 0.0477 ± 0.0165).

Abundance patterns in our data suggest priority effects. Vertically inherited ASVs gain higher abundance than those that are not inherited in both founding reproductives and offspring colony microbiomes (figure 4*a*; electronic supplementary material, figure S5). This implies that initial inoculation and microbial interactions may determine the propensity for ASVs to establish and proliferate. To shed light on community dynamics, we performed network analysis of the positive (co-occurrence of two ASVs) and negative (the presence of an ASV associated with the absence of another ASV) interactions [44]. Inheritance itself did not significantly affect microbial interactions (positive interactions linear model (LM): *F*_1,829_ = 0.9817, *p* = 0.3221; negative interactions LM: *F*_1,829_ = 1.468, *p* = 0.2260), implying that we cannot detect cooperation or competition between microbes. However, analysis of the network assortativity revealed that co-occurring ASVs tended to share the same mode of transmission (figure 4*b*). Assortativity was significantly higher than expected from the null model for ASVs with positive interactions (figure 4*c*), but not for disassortative negative interactions. Positive assortativity is often found among related microbes [44,45] and contributes to network structure. However, it may also reflect priority effects where co-occurring vertically transmitted ASVs direct the establishment of horizontally transmitted ASVs.

### (e) Microbial taxonomy and acquisition by founding reproductives drive inheritance

**Figure 5.**
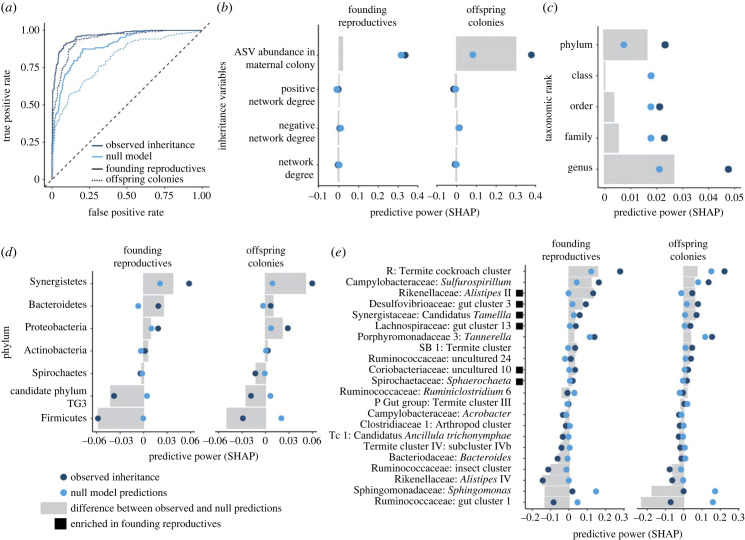
Random forest model predictions of bacterial inheritance. (*a*) The receiver operating characteristic plot indicated that variables related to observed inheritance (dark blue) hold greater predictive power than the null model (light blue). Transmission to founding reproductives (solid line) has greater predictive power for inheritance than transmission to offspring colonies (dotted line). (*b–e*) The predictive power (SHAP; Materials and methods) of variables related to inheritance was determined for observed inheritance and the null model, and grey bars indicate the difference between the two. Positive and negative values predict inheritance and non-inheritance, respectively. (*b*) ASV abundance in maternal colonies was a poor predictor of inheritance by founding reproductives compared to our null model, aligning with the results of figure 3. Conversely, ASV abundance in maternal colonies strongly predicted inheritance by workers in offspring colonies, signifying that ASVs re-establish in offspring colonies. Network degree did not affect inheritance. (*c–e*) Microbial taxonomy influenced the likelihood of inheritance, with phylum and genus being the best predictors, suggesting that both basal and recently evolved microbial traits play a role. (*e*) The 22 bacterial genera with the greatest difference in predictive power, including six genera that increased in abundance in founding reproductive guts (black squares; figure 3*b*); R, Ruminococcaceae; P, Porphyromonadaceae; TC 1, Termite_cluster_1 (electronic supplementary material, table S6).

We confirmed that vertical transmission by founding reproductives and ASV taxonomy strongly impact the probability of inheritance with an RF model. The predictive power of the RF model using the observed data was two- to 2.5-fold higher than the abundance-driven null model, highlighting the impact of variables aside from abundance; which was the only factor informing the null model (figure 5*a*). As expected, transmission to founding reproductives was more predictable than complete transmission to offspring colonies (figure 5*a*), where ASV abundance had little effect on transmission to founding reproductives but a strong effect on transmission to offspring colonies, aligning with (figure 3*c*) and indicating that vertically transmitted strains gain abundance in offspring colonies. Network degree, relating to the number of positive or negative interactions, did not have strong predictive power.

Phylum and genus were the most powerful taxonomic predictors of inheritance (figure 5*c*). Thus, an interplay between basal and more recently evolved traits affects inheritance. The largely anaerobic non-spore forming phyla Synergistetes [46], Bacteroidetes [47] and Proteobacteria [48] are strongly predicted to be represented in the inherited microbiome. Conversely, spore-formers like Actinobacteria and Firmicutes with more flexible respiration [49,50] are predicted to not be inherited. However, Candidate Phylum TG3 does not follow this pattern because although it is predicted to not be transmitted, it includes obligate anaerobes [47] (figure 5*d*), suggesting potential limitations to the model. Differential inheritance of bacterial phyla may be a consequence of their respective environmental resistance. These also reflect the relative importance of phyla, where Bacteroidetes encodes more carbohydrate-active enzymes than Firmicutes or Spirochaetes in fungus-farming termites [25]. Alternatively, they may reflect broader patterns of vertical transmission in animals. For example, we observed that Bacteroidetes has a higher propensity for vertical transmission, which has also been found in humans [38] and in mice [39]. Similarly, Firmicutes and spore formers are less-frequently transmitted and later colonizers of human microbiomes [37,51]. The genera with the strongest predictive power included six that were previously identified as differentially enriched in founding reproductives (figure 3*b*) and that are core gut microbiome members in fungus-farming termites [34].

## 3. Discussion

Extensive yet imperfect inheritance of the gut microbiome occurs in the termite superorganism. We assessed complete vertical transmission for the first time, by tracking individual bacterial taxa from parental colonies via founding reproductives to offspring colony workers. Aligned with our first hypothesis, we find that a consistent portion of the microbiome is inherited, in support of mixed-mode transmission. Our classification of bacteria to the ASV level at the strictest cut-off (a single SNP) allowed more detailed tracking and diversity assessments than OTU level assignments previously used [31,52]. Thus, by comparing ASV transmission across four distinct pedigrees, we uncovered transmission at the genus and ASV level that involves extensive pedigree specificity and biparental contributions. This builds on recent work [11,27,31], but demonstrates inheritance of a core community with unprecedented resolution, consistency across genetic pedigrees and biparental contributions of bacteria that persist in offspring colonies. In accordance with our second hypothesis, we found that founding reproductives have the greatest impact on shaping the inherited microbiome and secure transmission of a non-random set of bacteria. This results in pedigree-specific inheritance accomplished via selective enrichment of termite-specific and environmentally sensitive gut bacteria in founding reproductives.

The consistently inherited microbiota includes many bacteria that are co-adapted to fungus-farming termites [35,36] and present in developing and mature colonies [31,34]. The extent and patterns of inheritance were comparable across pedigrees, despite contributions from both founding reproductives. The large number of pedigree-specific ASVs supports that patterns arise as a consequence of vertical transmission [38,39]. Despite the relatively small subset of samples per pedigree, we documented congruent patterns in the numbers and genus origins of ASVs across pedigrees. While this indicates sufficient taxonomic resolution to detect inheritance, amplicon reads of such length only serve as an estimate of strain diversity [42]. Broad taxonomic patterns of vertical transmission align with key functions in the fungus-farming termite microbiome. For example, the phylum Bacteroidetes (Bacteroidota) and the family Campylobacteraceae are predicted to be inherited, and they maintain genes for carbohydrate-active enzymes or nitrogen recycling, respectively, in fungus-farming termites [25]. These functions may be particularly important during early stages of colony life, when nutrients are limited before the fungus comb is established. Strain-level metagenomics will be needed to clarify association specificity and to allow insights into the metabolic capacities of inherited symbionts.

The transfer of environmentally sensitive microbes is likely adaptive and critical to ensure that symbionts absent from the environment are reliably passed on from generation to generation. Founding reproductives serve as the only opportunity to do this, implying strong selection for reliable transmission, and mechanisms underlying these patterns should be explored further. Both founding reproductives and workers obtain their gut microbiome through oral and faecal trophallaxis [16]. However, the distinct phenotype and diet of reproductives, both of which are known to generally influence termite microbiomes [24], may contribute to selective enrichment of specific microbes. Reproductives are phenotypically distinct and highly plastic, as both their morphology [53,54] and gene expression [37] drastically change to meet shifting colony needs from founding to maturity. This extends to gut symbioses, as founding reproductives host over 80 bacterial genera at the time of colony founding, yet at maturity host only a few [40]. Founding reproductives are also presumed to have a different diet from workers to secure the energy stores needed for colony founding [55], then receive a unique diet as they mature [53]. Much like differently aged workers who consume distinct parts of the fungus comb that vary in nutritional composition [56,57] and reflect microbiome distinctions [58], any diet specific to founding reproductives should influence the establishing microbiota. Last, it remains to be established if mechanisms exist that allow reproductives to increase gut microbial loads before passing microbial inocula to workers, as was recently documented in non-Termitidae termites [59,60].

Despite enrichment, an individual founding reproductive only carries a portion of the parental colony microbiome; thus, biparental transmission should increase the probability of inheritance of key microbes. When leaving the parental colony, both male and female reproductives carry a subset of the parental microbiome [22,31]. Our findings indicate that biparental contributions result in an assembly of redundant and non-redundant strains. This is consistent with recent finding in *Coptotermes*, where each founding individual rarely transmitted the full set of protist species, but where their collective assemblies ensure high probability transmission of a critical set of protists [41]. Such bet hedging may suffice to ensure that bacterial symbionts absent from the environment are transmitted in fungus-farming and other termites.

Inheritance is completed when the first workers of the offspring colony obtain the diverse gut inoculum, yet the superorganismal microbiome will continue to develop as the colony matures. Inherited taxa appear to direct this development, potentially though priority effects. We only assessed the earliest stages in colony life before acquisition of the *Termitomyces* fungal cultivar [61] that complements the gut microbiome in symbiotic digestion of plant substrates [36]. Once the fungus garden establishes, acquisition of gut symbionts should ensue in ways that optimizes bacterial, termite and *Termitomyces* roles. The colony microbiome then continues to differentiate based on termite division of labour to support distinct roles and diets [40,62]. Once symbiotic homeostasis is accomplished for the termite superorganism, the bacterial community is in place to be passed onto future founding reproductives.

Consistent patterns in imperfect vertical transmission across colonies suggest that our results represent inheritance at the population level. These proximate patterns and association specificity may thus inform symbiotic evolution [6,7]. Indeed, our results align with termite–bacteria cladogenesis at evolutionary timescales, where lineages of bacterial symbionts exhibit distinct transmission modes, ranging from exclusive vertical transmission (strong co-cladogenesis) to predominant or exclusive horizontal transmission (lacking co-cladogenesis) [11,27]. Maturing colonies thus benefit from mixed-mode transmission that optimize tripartite associations between termite, fungus and bacterial microbiota at ecological timescales (microbial assembly) and over evolutionary time (extent of coevolution). Mixed-mode transmission evinced by the termite-bacterial symbiosis is emerging as the prevailing strategy to assemble adaptive microbiomes [10,63] when hosts remain in control over symbiont transmission between generations [64].

## 4. Materials and methods

### (a) Termite collection and pedigree establishment

In a previous study, four maternal colonies and one paternal colony of *Macrotermes natalensis* were sampled in Pretoria, South Africa in 2016 to establish four pedigrees (red, yellow, blue and green) [37]. Founding reproductives (alates) were crossed to establish offspring colonies. The parental colonies and founding reproductives were collected in the field, while the offspring colonies were established in the laboratory. Thus, there are three developmental time points—parental colonies, founding reproductives and offspring colonies [37]. These offspring colonies were sampled at three months old before they would naturally acquire their fungal cultivars [61]. From the offspring crosses, 6–10 colonies were used (yellow *n* = 9, red = 7, blue = 10 and green = 6). Termites from maternal colonies and female founding reproductives were collected when the crosses were established, while workers from the parental colony and male founding reproductives were sampled in 2018. This time lapse may in part explain the reduced observed paternal inheritance.

### (b) Gut dissections, DNA extraction and quantification of microbial load

Guts of reproductives were dissected aseptically and stored in RNAlater (Sigma-Aldrich, Germany), and workers and larvae were frozen. All samples were stored at −80°C. The termites from sterile castes were briefly rinsed in 70% EtOH and sterile dH_2_O (1 min each) to reduce surface contaminants and dissected aseptically. Worker, founding reproductive, and larvae samples represent pools of 5–10 guts, offspring queens 1–2 guts, parental kings single guts and parental queens were divided into three extractions due to tissue size and later pooled *in silico*. Offspring colony workers were pooled from two to three colonies within pedigrees due to small colony sizes. Termite gut samples, cellular mock community DNA standards (Zymobiomics, Nordic BioSite ApS, Denmark) and two negative controls were included for extraction and sequencing (electronic supplementary material, table S2). DNA was extracted using a modified DNeasy Blood and Tissue kit protocol (Qiagen, Germany) [35]. Relative microbial loads per termite gut were determined by quantifying copies of the V2 region of the 16S *rRNA* marker gene using droplet digital PCR (Bio-Rad, Denmark) [65].

### (c) Amplicon sequencing, quality control, taxonomic assignment and vertical transmission

DNA was sent to BGI-Hong Kong for paired-end HiSeq2500 amplicon sequencing with 341F/806R primers targeting the 16S *rRNA* gene V3–V4 region. Analyses were performed in R (v.3.6.1) [66]. We used the *dada2* pipeline (v.1.12.1) [67] with default parameters and the following adjustments to increase the stringency of the analysis: *truncLen* in *filterAndTrim* set to c(270, 260), *maxEE* to c(1,1) and truncQ to 1, and *minOverlap* in *mergePairs* to 20. We obtained a total of 50 016 ASVs in 4 403 068 non-chimeric paired-end sequences, of which 19 909 ASVs in 4 262 444 reads remained after removal of taxa with less than 25 reads across the full dataset.

We assigned ASVs with taxonomic ranks using default parameters of *assignTaxonomy* in *dada2*. Taxa were classified with the Dict_db v.3.0 database [68], after which ASVs without genus-level taxonomic assignments were reclassified with SILVA v.132 [69]. The cellular mock community validated the detection of all eight expected taxa.

Negative control samples lacked DNA and failed library preparation according to BGI-Hong Kong's sequencing service, suggesting very low levels of contaminants. The package *Decontam* was run to identify potential contaminant taxa (v.1.10.0) [70] despite the lack of negative controls, given that mature queens from parental colonies contained exceptionally low numbers of 16S rRNA copies (electronic supplementary material, table S2). We did not remove them from the dataset as: (i) we removed taxa with less than 25 reads, (ii) the majority of analyses were on workers and founding reproductives that carry high microbial loads minimizing any effect of contaminants, and (iii) the ASVs identified by *Decontam* represented only 1.3% total reads, yet some were taxa that are common in fungus-farming termites [34].

To be considered vertically transmitted, an ASV had to be found in the parental colony and all founding reproductives of that pedigree, and present in at least one offspring colony sample in the same pedigree. The number of ASVs, ASV abundance and the percentage of ASVs that were inherited were determined for each sample (electronic supplementary material, table S1).

### (d) Abundance-driven null model

To assess if the propensity for inheritance could be explained by ASV abundance, we simulated an abundance-driven null model. This model selected the same number of ASVs inherited by founding reproductives or offspring colonies within a termite pedigree from those in the respective maternal colony workers, weighed by their average relative abundance in these workers. We ran 100 iterations of the null model for each stage of inheritance and pedigree.

### (e) Network construction

We calculated ASV association networks for the offspring colony worker samples using the *SpiecEasi* package v.1.1.0 [44], the ‘mb’ algorithm, lambda.min.ratio = 1×10^−3^ and nlambda = 300. Prior to network calculation, we removed ASVs with less than 200 reads across all samples. To improve visualizations, we only included nodes with six or more connections. We handled graphs using *igraph* v.1.2.5, *network* v.1.16.0 and plotted them using *ggnet* v.0.1.0 and *ggnetwork* v.0.5.8. To understand ecological interactions related to inheritance, we calculated the assortativity coefficient for the variable ‘inheritance’ in *igraph*. This metric ranges from −1 indicating disassortativity (interactions between an inherited and a non-inherited ASVs), to 1 indicating assorativity (interactions between inherited ASVs or between non-inherited ASVs). We did this for the full networks and for subsets of networks with only positive or negative associations. For the null model, we calculated assortativity using the same network and assigned nodes to be inherited or non-inherited according to the results of the abundance-driven null model.

### (f) Statistical analyses

To assess increases and decreases in relative abundance of genera, we used *ALDEx2* (v. 1.24.0) [71], with the generalized LM function and pedigree as a fixed effect. We performed analyses that identified genus-level enrichment or depletions from maternal colony workers to founding reproductives, and from founding reproductives to offspring colony workers.

We then tested whether the relative abundance of an ASV within a genus predicts inheritance. ASVs were normalized by subtracting the mean relative abundance of the given genus from the relative abundance of the given ASV. First, we determined if abundance in maternal colony workers or founding reproductives impacted inheritance (figure 3*a*). We fitted a linear mixed model to predict whether ASVs were vertically transmitted or not with the transformed ASV abundances, while controlling for pedigree and genus as random effects. Second, we repeated this analysis with ASVs inherited by founding reproductives and offspring colony workers to determine if inheritance was related to vertical or non-vertical transmission (figure 4*a*). The models were run using the *lme* function in *nlme*. We created density plots to visualize the distribution of inherited versus environmentally acquired ASVs. The normalized abundance values were log-transformed as follows, *y*(*x*) = sign(*x*)*log(1 + abs(*x*)/10^−5^), to clarify contrasting distributions while the *x*-axis is labelled in accordance with the non-transformed values.

For our network, we tested if interactions could be explained by whether taxa were inherited or non-inherited, where positive and negative interactions indicate potential cooperation and competition. To do this, we created an LM where all interactions were the response variables and the fixed effects of ASV inheritance/non-inheritance, the log of ASV abundance and their interaction. The latter was not significant and was consequently removed from the final model.

We used permutation tests to measure any statistical difference between patterns of observed inheritance and the null model. First, we performed this for the number of genera vertically transmitted to founding reproductives and offspring colonies within each pedigree. Second, we did this for the assortativity coefficient of inheritance for positive and negative network interactions. The permutation was manually calculated by determining the proportion of simulations above or below the observed value. For example, if 96 of the 100 iterations of the simulation were above the observed value, *p* was 0.04.

### (g) Random forest models to identify major features predicting inheritance

We fitted RF classifiers implemented in Python's scikit-learn to uncover which experimental characteristics predict inheritance of an ASV, focusing on the impact of bacterial taxonomy, abundance in maternal colonies, and network features. This was done in six different models based on dependent variables: observed and null model inheritance to founding reproductives, offspring colonies, and overall. Each ASV was classified eight times (once per stage of inheritance and per pedigree) with each taxonomical level recorded (the null model was assigned a dummy binary variable), which, along with stage of inheritance, network features and abundance (normalized as above), were the independent variables. ASVs containing missing data in any features were removed.

The contribution of each predictor to inheritance was estimated with Gini importance and Shapley Additive exPlanations (SHAP) [72] values. We randomly split each dataset into a training set of 80% and a test set of 20% of the ASVs. We then used the training set to form a 10-fold cross-validated grid search for the best number of features used in each split, while fixing the number of trees to 500. Using the best model, we predicted the test's ASV inheritance status. We used a game theory approach to unlock the predictive potential of properties the experimental features for each ASV via SHAP values.

To compare feature importance between the null models and the observed inheritance models, we normalized SHAP values from each model by retrieving the difference between the SHAP values for binomial predictors (true/false) and the difference between predictor values above and below the median for continuous predictors. Predictive power ratios were calculated to determine the overall power of the different models (figure 5*a*). Power ratios were calculated as the sum of the absolute values of the SHAP of each feature from the observed data divided by that of the null model. We then calculated the difference between the normalized SHAP of the observed inheritance model minus the null model. Positive SHAP predict vertical transmission and negative values predict horizontal transmission.

## Data Availability

Amplicon sequences are available from the SRA archive in GenBank (BioProject PRJNA860052). ASV tables, taxonomy, metadata and ASVs identified as potential contaminants are available through Figshare (project: Extensive inheritance of gut microbial communities in a superorganismal termite, dois: https://doi.org/10.6084/m9.figshare.22047479.v1 [73], https://doi.org/10.6084/m9.figshare.22047467.v1 [74], https://doi.org/10.6084/m9.figshare.22047470.v3 [75], https://doi.org/10.6084/m9.figshare.22047539.v1 [76]). Code for analyses is available at https://doi.org/10.5281/zenodo.8321616 [77]. The data are provided in the electronic supplementary material [78].

## References

[1] Engel P, Moran NA. 2013 The gut microbiota of insects—diversity in structure and function. FEMS Microbiol. Rev. **37**, 699-735. (10.1111/1574-6976.12025) 23692388

[2] Maynard CL, Elson CO, Hatton RD, Weaver CT. 2012 Reciprocal interactions of the intestinal microbiota and immune system. Nature **489**, 231-241. (10.1038/nature11551) 22972296 PMC4492337

[3] Rowland I, Gibson G, Heinken A, Scott K, Swann J, Thiele I, Tuohy K. 2018 Gut microbiota functions: metabolism of nutrients and other food components. Eur. J. Nutr. **57**, 1-24. (10.1007/s00394-017-1445-8) PMC5847071 28393285

[4] Bright M, Bulgheresi S. 2010 A complex journey: transmission of microbial symbionts. Nat. Rev. Microbiol. **8**, 218-230. (10.1038/nrmicro2262) 20157340 PMC2967712

[5] Coyte KZ, Rao C, Rakoff-Nahoum S, Foster KR. 2021 Ecological rules for the assembly of microbiome communities. PLoS Biol. **19**, e3001116. (10.1371/journal.pbio.3001116) 33606675 PMC7946185

[6] Frank SA. 1997 Models of symbiosis. Am. Nat. **150**(Suppl. 1), S80-S99. (10.1086/286051) 18811314

[7] Yamamura N. 1993 Vertical transmission and evolution of mutualism from parasitism. Theor. Popul. Biol. **44**, 95-109. (10.1006/tpbi.1993.1020)

[8] Kiers ET, West SA. 2015 Evolving new organisms via symbiosis. Science **348**, 392-394. (10.1126/science.aaa9605) 25908807

[9] Bjork JR, Diez-Vives C, Astudillo-Garcia C, Archie EA, Montoya JM. 2019 Vertical transmission of sponge microbiota is inconsistent and unfaithful. Nat. Ecol. Evol. **3**, 1172. (10.1038/s41559-019-0935-x) 31285574 PMC6914380

[10] Leftwich PT, Edgington MP, Chapman T. 2020 Transmission efficiency drives host–microbe associations. Proc. R. Soc. B **287**, 20200820. 10.1098/rspb.2020.0820 PMC7542779 32873208

[11] Bourguignon T, Lo N, Dietrich C, Sobotnik J, Sidek S, Roisin Y, Brune A, Evans TA. 2018 Rampant host switching shaped the termite gut microbiome. Curr. Biol. **28**, 649-654.e2. (10.1016/j.cub.2018.01.035) 29429621

[12] Kwong WK, Medina LA, Koch H, Sing K-W, Soh EJY, Ascher JS, Jaffe R, Moran NA. 2017 Dynamic microbiome evolution in social bees. Sci. Adv. **3**, e1600513. (10.1126/sciadv.1600513) 28435856 PMC5371421

[13] Sanders JG, Powell S, Kronauer DJ, Vasconcelos HL, Frederickson ME, Pierce NE. 2014 Stability and phylogenetic correlation in gut microbiota: lessons from ants and apes. Mol. Ecol. **23**, 1268-1283. (10.1111/mec.12611) 24304129

[14] Boomsma JJ, Gawne R. 2018 Superorganismality and caste differentiation as points of no return: how the major evolutionary transitions were lost in translation. Biol. Rev. Camb. Phil. Soc. **93**, 28-54. (10.1111/brv.12330) 28508537

[15] Lanan MC, Rodrigues PA, Agellon A, Jansma P, Wheeler DE. 2016 A bacterial filter protects and structures the gut microbiome of an insect. ISME J. **10**, 1866-1876. (10.1038/ismej.2015.264) 26872040 PMC5029173

[16] Nalepa CA. 2015 Origin of termite eusociality: trophallaxis integrates the social, nutritional, and microbial environments. Ecol. Entomol. **40**, 323-335. (10.1111/een.12197)

[17] Powell JE, Martinson VG, Urban-Mead K, Moran NA. 2014 Routes of acquisition of the gut microbiota of the honey bee *Apis mellifera*. Appl. Environ. Microbiol. **80**, 7378-7387. (10.1128/AEM.01861-14) 25239900 PMC4249178

[18] Hu Y et al. 2023 Partner fidelity and environmental filtering preserve stage-specific turtle ant gut symbioses for over 40 million years. Ecol. Monogr. **93**, e1560. (10.1002/ecm.1560)

[19] Meirelles LA, McFrederick QS, Rodrigues A, Mantovani JD, de Melo Rodovalho C, Ferreira H, Bacci M, Mueller UG. 2016 Bacterial microbiomes from vertically transmitted fungal inocula of the leaf-cutting ant *Atta texana*. Environ. Microb. Rep. **8**, 630-640. (10.1111/1758-2229.12415) 27273758

[20] Su Q et al. 2021 Strain-level analysis reveals the vertical microbial transmission during the life cycle of bumblebee. Microbiome **9**, 216. (10.1186/s40168-021-01163-1) 34732245 PMC8567698

[21] Diouf M, Herve V, Mora P, Robert A, Frechault S, Rouland-Lefevre C, Miambi E. 2018 Evidence from the gut microbiota of swarming alates of a vertical transmission of the bacterial symbionts in *Nasutitermes arborum* (Termitidae, Nasutitermitinae). Antonie Van Leeuwenhoek **111**, 573-587. (10.1007/s10482-017-0978-4) 29127624

[22] Michaud C, Herve V, Dupont S, Dubreuil G, Bezier AM, Meunier J, Brune A, Dedeine F. 2020 Efficient but occasionally imperfect vertical transmission of gut mutualistic protists in a wood-feeding termite. Mol. Ecol. **29**, 308-324. (10.1111/mec.15322) 31788887

[23] Bucek A, Sobotnik J, He S, Shi M, McMahon DP, Holmes EC, Roisin Y, Lo N, Bourguignon T. 2019 Evolution of termite symbiosis informed by transcriptome-based phylogenies. Curr. Biol. **29**, 3728-3734 e4. (10.1016/j.cub.2019.08.076) 31630948

[24] Brune A. 2014 Symbiotic digestion of lignocellulose in termite guts. Nat. Rev. Microbiol. **12**, 168-180. (10.1038/nrmicro3182) 24487819

[25] Arora J et al. 2022 The functional evolution of termite gut microbiota. Microbiome **10**, 78. (10.1186/s40168-022-01258-3) 35624491 PMC9137090

[26] Desai MS, Strassert JF, Meuser K, Hertel H, Ikeda-Ohtsubo W, Radek R, Brune A. 2010 Strict cospeciation of devescovinid flagellates and Bacteroidales ectosymbionts in the gut of dry-wood termites (Kalotermitidae). Environ. Microbiol. **12**, 2120-2132. (10.1111/j.1462-2920.2009.02080.x) 21966907

[27] Arora J et al. 2023 Evidence of cospeciation between termites and their gut bacteria on a geological time scale. Proc. R. Soc. B **290**, 20230619. (10.1098/rspb.2023.0619) PMC10281810 37339742

[28] da Costa RR, Poulsen M. 2018 Mixed-mode transmission shapes termite gut community assemblies. Trends Microbiol. **26**, 557-559. (10.1016/j.tim.2018.04.005) 29752168

[29] Benjamino J, Graf J. 2016 Characterization of the core and caste-specific microbiota in the termite, *Reticulitermes flavipes*. Front. Microbiol. **7**, 171. (10.3389/fmicb.2016.00171) 26925043 PMC4756164

[30] Chouvenc T, Su NY. 2017 Irreversible transfer of brood care duties and insights into the burden of caregiving in incipient subterranean termite colonies. Ecol. Entomol. **42**, 777-784. (10.1111/een.12443)

[31] Diouf M et al. 2023 Succession of the microbiota in the gut of reproductives of *Macrotermes subhyalinus* (Termitidae) at colony foundation gives insights into symbionts transmission. Front. Ecol. Evol. **10**, 1055382. (10.3389/fevo.2022.1055382)

[32] Dietrich C, Kohler T, Brune A. 2014 The cockroach origin of the termite gut microbiota: patterns in bacterial community structure reflect major evolutionary events. Appl. Environ. Microbiol. **80**, 2261-2269. (10.1128/AEM.04206-13) 24487532 PMC3993134

[33] Otani S, Hansen LH, Sorensen SJ, Poulsen M. 2016 Bacterial communities in termite fungus combs are comprised of consistent gut deposits and contributions from the environment. Microb. Ecol. **71**, 207-220. (10.1007/s00248-015-0692-6) 26518432 PMC4686563

[34] Otani S et al. 2014 Identifying the core microbial community in the gut of fungus-growing termites. Mol. Ecol. **23**, 4631-4644. (10.1111/mec.12874) 25066007

[35] Hu HF, da Costa RR, Pilgaard B, Schiott M, Lange L, Poulsen M. 2019 Fungiculture in termites is associated with a mycolytic gut bacterial community. Msphere **4**, 10-128. 10.1128/mSphere.00165-19 PMC6520439 31092601

[36] Poulsen M et al. 2014 Complementary symbiont contributions to plant decomposition in a fungus-farming termite. Proc. Natl Acad. Sci. USA **111**, 14 500-14 505. (10.1073/pnas.1319718111) PMC4209977 25246537

[37] Séité S et al. 2022 Lifespan prolonging mechanisms and insulin upregulation without fat accumulation in long-lived reproductives of a higher termite. Commun. Biol. **5**, 44. (10.1038/s42003-021-02974-6) 35027667 PMC8758687

[38] Olm MR et al. 2022 Robust variation in infant gut microbiome assembly across a spectrum of lifestyles. Science **376**, 1220-1223. (10.1126/science.abj2972) 35679413 PMC9894631

[39] Moeller AH, Suzuki TA, Phifer-Rixey M, Nachman MW. 2018 Transmission modes of the mammalian gut microbiota. Science **362**, 453-457. (10.1126/science.aat7164) 30361372

[40] Otani S, Zhukova M, Kone NA, da Costa RR, Mikaelyan A, Sapountzis P, Poulsen M. 2019 Gut microbial compositions mirror caste-specific diets in a major lineage of social insects. Environ. Microb. Rep. **11**, 196-205. (10.1111/1758-2229.12728) PMC6850719 30556304

[41] Velenovsky JF, De Martini F, Hileman JT, Gordon JM, Su NY, Gile GH, Chouvenc T. 2023 Vertical transmission of cellulolytic protists in termites is imperfect, but sufficient, due to biparental transmission. Symbiosis **90**, 25-38. (10.1007/s13199-023-00917-9)

[42] Strube ML. 2021 RibDif: can individual species be differentiated by 16S sequencing? Bioinf. Adv. **1**, vbab020. (10.1093/bioadv/vbab020) PMC9710640 36700109

[43] Debray R, Herbert RA, Jaffe AL, Crits-Christoph A, Power ME, Koskella B. 2022 Priority effects in microbiome assembly. Nat. Rev. Microbiol. **20**, 109-121. (10.1038/s41579-021-00604-w) 34453137

[44] Kurtz ZD, Muller CL, Miraldi ER, Littman DR, Blaser MJ, Bonneau RA. 2015 Sparse and compositionally robust inference of microbial ecological networks. PLoS Comput. Biol. **11**, e1004226. (10.1371/journal.pcbi.1004226) 25950956 PMC4423992

[45] Hall CV, Lord A, Betzel R, Zakrzewski M, Simms LA, Zalesky A, Radford-Smith G, Cocchi L. 2019 Co-existence of network architectures supporting the human gut microbiome. iScience **22**, 380-391. (10.1016/j.isci.2019.11.032) 31812808 PMC6911941

[46] Jumas-Bilak E, Marchandin H. 2014 The phylum synergistetes. In The prokaryotes: other major lineages of bacteria and the archaea (eds E Rosenberg, EF DeLong, S Lory, E Stackebrandt, F Thompson), pp. 931-954. Berlin, Germany: Springer.

[47] Krieg JTS NR, Brown DR, Hedlund BP, Paster BJ, Ward NL, Ludwig W, Whitman WB. 2010 Bergey's manual of systematic bacteriology. New York, NY: Springer.

[48] Beskrovnaya P, Fakih D, Morneau I, Hashimi A, Bello DG, Xing SP, Nanci A, Huan T, Tocheva EI. 2021 No endospore formation confirmed in members of the phylum proteobacteria. Appl. Environ. Microbiol. **87**, e02312-20. (10.1128/AEM.02312-20) 33355101 PMC8090866

[49] Whitman WB MG, Kämpfer P, Busse H-J, Trujillo ME, Ludwig W, Suzuki K. 2012 Bergey's manual of systematic bacteriology. New York, NY: Springer.

[50] Vos P, Garrity G, Jones D, Krieg NR, Ludwig W, Rainey FA, Schleifer K, Whitman WB. 2009 Bergey's manual of systematic bacteriology. New York, NY: Springer.

[51] Egan M, Dempsey E, Ryan CA, Ross RP, Stanton C. 2021 The sporobiota of the human gut. Gut Microbes **13**, 1-17. (10.1080/19490976.2020.1863134) PMC7801112 33406976

[52] Callahan BJ, McMurdie PJ, Holmes SP. 2017 Exact sequence variants should replace operational taxonomic units in marker-gene data analysis. Isme J. **11**, 2639-2643. (10.1038/ismej.2017.119) 28731476 PMC5702726

[53] Tasaki E et al. 2023 The royal food of termites shows king and queen specificity. PNAS Nexus **2**, pgad222. (10.1093/pnasnexus/pgad222) 37457894 PMC10338896

[54] Eggleton P. 2010 An introduction to termites: biology, taxonomy and functional morphology. In Biology of termites: a modern synthesis (eds DE Bignell, Y Roisin, N Lo), pp. 1-26. Berlin, Germany: Springer.

[55] Lo N, Tokuda G, Watanabe H. 2010 Evolution and function of endogenous termite cellulases. In: Bignell DE, Roisin Y, Lo N. Biology of termites: a modern synthesis, pp. 51-67. (10.1007/978-90-481-3977-4_3)

[56] da Costa RR et al. 2018 Enzyme activities at different stages of plant biomass decomposition in three species of fungus-growing termites. Appl. Environ. Microbiol. **84**, e01815-17. (10.1128/AEM.01815-17) 29269491 PMC5812949

[57] Li H, Yang M, Chen Y, Zhu N, Lee CY, Wei JQ, Mo J. 2015 Investigation of age polyethism in food processing of the fungus-growing termite *Odontotermes formosanus* (Blattodea: Termitidae) using a laboratory artificial rearing system. J. Econ. Entomol. **108**, 266-273. (10.1093/jee/tou005) 26470129

[58] Li H et al. 2016 Age polyethism drives community structure of the bacterial gut microbiota in the fungus-cultivating termite *Odontotermes formosanus*. Environ. Microbiol. **18**, 1440-1451. (10.1111/1462-2920.13046) 26346907

[59] Velenovsky J, Gile G, Su N-Y, Chouvenc T. 2021 Dynamic protozoan abundance of *Coptotermes* kings and queens during the transition from biparental to alloparental care. Insectes Soc. **68**, 33-40. (10.1007/s00040-021-00808-6)

[60] Inagaki T, Matsuura K. 2016 Colony-dependent sex differences in protozoan communities of the lower termite *Reticulitermes speratus* (Isoptera: Rhinotermitidae). Ecol. Res. **31**, 749-755. (10.1007/s11284-016-1387-2)

[61] Aanen DK, Eggleton P, Rouland-Lefevre C, Guldberg-Froslev T, Rosendahl S, Boomsma JJ. 2002 The evolution of fungus-growing termites and their mutualistic fungal symbionts. Proc. Natl Acad. Sci. USA **99**, 14 887-14 892. (10.1073/pnas.222313099) PMC137514 12386341

[62] Sinotte VM, Renelies-Hamilton J, Taylor BA, Ellegaard KM, Sapountzis P, Vasseur-Cognet M, Poulsen M. 2020 Synergies between division of labor and gut microbiomes of social insects. Front. Ecol. Evol. **7**, 503. (10.3389/fevo.2019.00503)

[63] Bruijning M, Henry LP, Forsberg SKG, Metcalf CJE, Ayroles JF. 2021 Natural selection for imprecise vertical transmission in host–microbiota systems. Nature Ecol. Evol. **6**, 77-87. (10.1038/s41559-021-01593-y) 34949814 PMC9901532

[64] Foster KR, Schluter J, Coyte KZ, Rakoff-Nahoum S. 2017 The evolution of the host microbiome as an ecosystem on a leash. Nature **548**, 43-51. (10.1038/nature23292) 28770836 PMC5749636

[65] Zhukova M, Sapountzis P, Schiott M, Boomsma JJ. 2017 Diversity and transmission of gut bacteria in *Atta* and *Acromyrmex* leaf-cutting ants during development. Front. Microbiol. **8**, 1942. (10.3389/fmicb.2017.01942) 29067008 PMC5641371

[66] R Core Team. 2017 R: a language and environment for statistical computing. Vienna, Austria: R Foundation for Statistical Computing.

[67] Callahan BJ, McMurdie PJ, Rosen MJ, Han AW, Johnson AJA, Holmes SP. 2016 DADA2: high-resolution sample inference from Illumina amplicon data. Nat. Methods **13**, 581. (10.1038/nmeth.3869) 27214047 PMC4927377

[68] Mikaelyan A, Kohler T, Lampert N, Rohland J, Boga H, Meuser K, Brune A. 2015 Classifying the bacterial gut microbiota of termites and cockroaches: a curated phylogenetic reference database (DictDb). Syst. Appl. Microbiol. **38**, 472-482. (10.1016/j.syapm.2015.07.004) 26283320

[69] Quast C, Pruesse E, Yilmaz P, Gerken J, Schweer T, Yarza P, Peplies J, Glöckner FO. 2013 The SILVA ribosomal RNA gene database project: improved data processing and web-based tools. Nucleic Acids Res. **41**(D1), D590-D596. (10.1093/nar/gks1219) 23193283 PMC3531112

[70] Davis NM, Proctor DM, Holmes SP, Relman DA, Callahan BJ. 2018 Simple statistical identification and removal of contaminant sequences in marker-gene and metagenomics data. Microbiome **6**, 1-4. (10.1186/s40168-018-0605-2) 30558668 PMC6298009

[71] Gloor GB, Macklaim JM, Fernandes AD. 2016 Displaying variation in large datasets: plotting a visual summary of effect sizes. J. Comput. Graph Stat. **25**, 971-979. (10.1080/10618600.2015.1131161)

[72] Lundberg SM et al. 2020 From local explanations to global understanding with explainable AI for trees. Nat. Mach. Intell. **2**, 56-67. (10.1038/s42256-019-0138-9) 32607472 PMC7326367

[73] Sinotte V. 2023 Taxonomy of ASVs. Figshare. (10.6084/m9.figshare.22047479.v1)

[74] Sinotte V. 2023 Relative abundance of ASVs. Figshare. (10.6084/m9.figshare.22047467.v1)

[75] Sinotte V. 2023 Metadata and 16S gene quantification. Figshare. (10.6084/m9.figshare.22047470.v3)

[76] Sinotte V. 2023 ASVs identified as potential contaminants. Figshare. (10.6084/m9.figshare.22047539.v1)

[77] vsin632. 2023 vsin632/vertical_transmission:vertical.transmission.v2. Zenodo. (10.5281/zenodo.8321616)

[78] Sinotte VM, Renelies-Hamilton J, Andreu-Sánchez S, Vasseur-Cognet M, Poulsen M. 2023 Selective enrichment of founding reproductive microbiomes allows extensive vertical transmission in a fungus-farming termite. Figshare. (10.6084/m9.figshare.c.6845629) PMC10581767 37848067

